# High-Throughput Screening Identifies Proteasome Inhibitors as Potentiators of Type III Interferon JAK-STAT Signaling and Antiviral Immunity

**DOI:** 10.64898/2026.10.12.758093

**Published:** 2026-10-19

**Authors:** Jean-Patrick Parisien, Crystal O’Leary, Shima Shabani, Sara Fernandez-Dunne, Laura T. Huynh, Aleksandra K. Kurowska, Curt M. Horvath, Juan L. Mendoza

## Abstract

Type III interferon (IFN3, IFNλ) drives antiviral defense at epithelial barriers with a more favorable safety profile than Type I interferon (IFN1), but weaker signaling limits its therapeutic potential. To identify small molecules that potentiate IFN-JAK-STAT signaling, we developed a high-throughput live-cell assay measuring STAT1/STAT2 phosphorylation in A549 cells and screened ∼8,640 compounds. This identified proteasome inhibitors (MG132, delanzomib, ixazomib, oprozomib, bortezomib) as selective potentiators of IFN3-induced STAT activation, with minimal effect on IFN1 signaling. Proteasome inhibition enhanced IFN3 antiviral activity to levels approaching IFN1. Mechanistically, proteasome inhibition increased cell-surface IL10Rβ, the limiting IFN3 receptor subunit, thereby increasing receptor JAK-STAT signaling. It did not alter IFN1 receptor levels but protected IFNAR2 from late ligand-induced downregulation. These findings establish receptor subunit availability as a rate-limiting, druggable node governing IFN3 signaling strength and demonstrate a scalable platform for identifying cytokine-selective signaling potentiators, with implications for mucosal antiviral therapeutics.

## Full Text Availability

The license terms selected by the author(s) for this preprint version do not permit archiving in PMC. The full text is available from the preprint server.

